# Characterisation of the Plasmidome within *Enterococcus faecalis* Isolated from Marginal Periodontitis Patients in Norway

**DOI:** 10.1371/journal.pone.0062248

**Published:** 2013-04-30

**Authors:** Xiaobo Song, Jinglu Sun, Theresa Mikalsen, Adam P. Roberts, Arnfinn Sundsfjord

**Affiliations:** 1 Department of Medical Biology, Faculty of Health Sciences, University of Tromsø, Tromsø, Norway; 2 Department of Microbial Diseases, UCL Eastman Dental Institute, University College London, London, United Kingdom; 3 Department of Microbiology and Infection Control, Reference Centre for Detection of Antimicrobial Resistance (K-res), University Hospital of Northern Norway, Tromsø, Norway; The University of Hong Kong, Hong Kong

## Abstract

The present study aimed to identify and characterize plasmids in a national collection of oral *Enterococcus faecalis* (n = 106) isolated from patients with marginal periodontitis. Plasmid replicon typing was performed by multiplex-PCR and sequencing with specific primers for 18 *rep-*families and 1 unique sequence. Additional plasmid analysis by S1-PFGE was performed for comparison. Totally 120 plasmid replicon amplicons of seven *rep*-families were identified in 93 *E. faecalis* strains, e.g. *rep*9 (prototype pCF10), *rep*6 (prototype pS86), *rep*2 (prototype pRE25/pEF1), and *rep*8 (prototype pAM373). *Rep*9 was the most predominant *rep*-family being detected in 81 (76.4%) strains. Forty of these strains were tetracycline resistant and three were erythromycin resistant. *Rep*6 was the second predominant *rep*-family being detected in 22 (20.8%) strains. *Rep*2 was detected in eight (7.5%) strains. All *rep*2-positive strains were resistant to tetracycline and/or erythromycin and six of them contained Tn*916*/Tn*1545* genes. The *rep*-positive *E. faecalis* exhibited divergence in multilocus sequence types (STs). There was a significant correlation between *rep*9 and ST21, while multiple *rep*-families appeared in ST40. Totally 145 plasmid bands were identified in 95 *E. faecalis* strains by S1-PFGE, 59 strains carrying one plasmid, 27 carrying two, five carrying three, three carrying four, and one strain carrying five plasmids. Plasmid sizes varied between 5–150 kbp. There was a significant correlation between the number of plasmids identified by PCR *rep*-typing and by S1-PFGE. The results indicate that the majority of *E. faecalis* of marginal periodontitis are likely to be a reservoir for diverse mobile genetic elements and associated antimicrobial resistance determinants.

## Introduction

The oral microflora is an important reservoir for transferable antimicrobial resistance [1], [2], [3], [4]. Our previous study of oral *Enterococcus faecalis* isolated from Norwegian patients with marginal and apical periodontitis showed that approximately 50% of the strains were resistant to one or more of the tested antimicrobial agents, mostly tetracycline and/or erythromycin [5], [6]. These observations are consistent with former studies showing resistance towards commonly used antimicrobials in enterococci isolated from the oral cavity [7], [8], [9]. Enterococci are adept at acquiring antimicrobial resistance, both by point mutations and horizontal gene transfer [10], [11]. Intercellular transfer of antimicrobial resistance determinants in enterococci has been associated with numerous mobile genetic elements (MGEs) including conjugative plasmids and conjugative transposons [10], [12]. In *E. faecalis* the narrow host-range, pheromone-responsive, conjugative plasmids and the Tn*916*/Tn*1545*-like conjugative transposons that can be found both on these plasmids (e.g. pCF10 contains Tn*916*) and integrated into the chromosome, have been shown to confer resistance to tetracycline and/or erythromycin [13], [14].

A number of schemes have been developed for plasmid classification, for example plasmid host range, integrative incompatibility, plasmid DNA fingerprinting, replicon typing, and plasmid sequencing [15]. Recently, a PCR-based system has been established for classifying plasmids from enterococci and other Gram-positive bacteria by targeting specific replicon initiation genes (*rep*) of plasmid DNA [16]. To our knowledge plasmid *rep*-types have not been systematically investigated in large collections of oral enterococcal isolates. The aims of the present study were to identify and characterize plasmid replicons and the overall plasmid contents in our national collection of more than one hundred clinical oral *E. faecalis,* and to explore the association of plasmids and conjugative transposons with respect to the previously observed multilocus sequence types (STs) and resistance phenotypes.

## Materials and Methods

### Ethics statement

This study is a laboratory identification of mobile genetic elements from *E. faecalis* strains grown in artificial media. The study does not involve the sample collection or patient data, and no patient intervention occurred with the obtained results.

### Bacterial strains

A total of 106 *E. faecalis* strains were obtained from our previous study [6]. The 19 control strains containing plasmids representing 18 unique *rep*-families and one unique *rep*-sequence were used as positive controls in the recently described PCR-based plasmid *rep*-typing system [16]. The two reference strains *E. faecalis* DS16 harbouring pAD1 (58 kb) and pAD2 (25 kb) and *E. faecalis* OG1X harbouring pCF10 (67.7 kb) were used as positive controls in the S1-nuclease pulsed-field gel electrophoresis (PFGE) assay.

### Plasmid replicon typing by multiplex PCR and sequencing

Whole bacterial DNA was extracted according to the boiling lysis protocol [17]. Molecular identification of *E. faecalis* was preformed by PCR with species-specific primers complementary to internal regions of *E. faecalis* 16S rRNA [18]. Six multiplex-PCRs with the specific primers for 18 *rep-*families and 1 single PCR for 1 unique sequence were used for plasmid classification: *rep*1 (prototype pIP501), *rep*2 (prototype pRE25/pEF1), *rep*3 (prototype pAW63), *rep*4 (prototype pMBB1), *rep*5 (prototype pN315), *rep*6 (prototype pS86), *rep*7 (prototype pUSA02), *rep*8 (prototype pAM373), *rep*9 (prototype pCF10), *rep*10 (prototype pIM13), *rep*11 (prototype pEF1071), *rep*13 (prototype pC194), *rep*14 (prototype pRI1), *rep*15 (prototype pUSA03), *rep*16 (prototype pSAS), *rep*17 (prototype pRUM), *rep*18 (prototype pEF418), *rep*19 (prototype pUB101) and the unique sequence of pMG1 [16]. After purification with EXO-SAPIT (GE Healthcare, Oslo, Norway) the selected PCR amplicons were sequenced by an ABI3130XL 20 genetic analyzer (Applied Biosystems) with the Big Dye® v 3.1 cycle sequencing kit [19]. DNA sequences were aligned and analyzed by the Bioedit software v7.05 (Ibis Therapeutics, Carlsbad, CA 92008), and the sequence identity of each replicon was determined by comparison with the respective *rep*-family control sequence.

### Plasmid analyses by S1-PFGE

A plasmid analysis by S1-PFGE was performed according to Rosvoll et al. [20]. In brief total DNA embedded in agarose gel plug was treated with 20 U of S1 nuclease (Takara, AH Diagnostics, Oslo, Norway) and separated by pulsed-field gel electrophoresis on CHEF-DR® III device (Bio-Rad, Hercules, CA. USA). Each band represented a linear plasmid. Low Range PFG Marker (New England Biolabs) ranging from 4.36 – 194 kbp was used as the molecular weight marker.

### Detection of Tn*916*/Tn*1545* genes by PCR

Since the *rep*9 (pCF10) contains a copy of the conjugative transposon Tn*916*, we decided to detect the presence of genes present on these MGEs in addition to the plasmid replicon. Genes present on Tn*916*-like elements were detected by specific PCRs as previously described targeting the genes for tetracycline resistant [*tet*(M)], erythromycin resistance [*erm*(B)] and the integrase gene [*intTn*] [21], [22]. PCR products were verified by agarose gel electrophoresis and selected amplicons were sequenced for confirmation.

### Statistical analysis

SPSS 16.0 for windows was used for statistical analysis. The Pearson’s χ^2^ test was used to examine correlation between the replicon number by *rep*-typing PCR and the plasmid number by S1-PFGE. The Fisher’s exact probability test was applied for calculating the Phi coefficient of association between *rep*9 and ST21. *p*<0.05 was considered significant.

## Results

### Plasmid detection and classification by replicon typing and S1-PFGE

A total of 120 plasmid replicon amplicons were identified in 93 (87.7%) oral *E. faecalis* strains by PCR. Seven *rep-*families were determined among these plasmid replicons (Table 1), while *rep*3, *rep*4, *rep*5, *rep*10, *rep*11, *rep*13, *rep*14, *rep*15, *rep*16, *rep*18, *rep*19 and the unique *rep* on pMG1 were not detected. A single *rep*-family was detected in 68 strains, two *rep*-families were detected in 24, and three *rep*-families detected in one. *Rep*9 (pCF10) was the most predominant *rep*-family, being identified in 81 (76.4%) strains from marginal periodontitis. Forty of these strains were tetracycline resistant and three were erythromycin resistant [6]. *Rep*6 (pS86) was the second most predominant *rep*-family being detected in 22 (20.8%) strains. *Rep*2 (pRE25) was third most predominant *rep*-family detected in eight strains, all of which were resistant to tetracycline and/or erythromycin. *Rep*8 was detected in four strains and all of them were resistant to tetracycline. The *rep*-positive *E. faecalis* exhibited divergence in multilocus STs that were derived from our previous study [6]. There was a significant correlation between *rep*9 and ST21 (*p* = 0.01), while multiple *rep*-families appeared in ST40.

**Table 1:** Distribution of replicon types, antibiotic resistance, Tn*916/*Tn*1545* associated genes and multi-locus sequence types among 106 *E. faecalis* in marginal periodontitis.

Strain			Plasmid		Tn*916* gene	Resistance*
Sequence type*	Number		*Rep*-family	Prototype		
4	1		6, 9	pCF10, pS86	–	–
16	5	1	9	pCF10	–	–
		1	9	pCF10	*tet*(M), *intTn*	tetracycline
		1	–	–	*tet(M)*, *intTn*	tetracycline
		1	–	–	*tet(M)*, *erm*(B), *intTn*	tetracycline, erythromycin
		1	–	–	*tet(M)*, *erm*(B), *intTn*	tetracycline, erythromycin, gentamicin
21	19	15	9	pCF10	*tet(M)*, *intTn*	tetracycline
		1	9	pCF10	–	–
		2	6, 9	pCF10, pS86	*tet(M)*, *intTn*	tetracycline
		1	6, 9	pCF10, pS86	–	–
25	3	1	6, 9	pCF10, pS86	–	–
		1	–	–	–	–
		1	–	–	*tet(M)*, *intTn*	tetracycline
30	7	1	9	pCF10	–	–
		1	9	pCF10	*tet(M)*	tetracycline, trimethoprim
		1	9	pCF10	*tet(M)*, *intTn*	tetracycline
		1	9	pCF10	–	tetracycline
		1	6, 9	pCF10, pS86	–	–
		1	9, 17	pCF10, pRUM	–	–
		1	––	–	–	–
34	1		9	pCF10	–	–
35	1	1	1	pIP501	*tet(M)*, *erm*(B), *intTn*	tetracycline, erythromycin, gentamicin
40	10	1	9	pCF10	–	–
		1	9	pCF10	*tet(M)*, *intTn*	tetracycline
		1	2	pRE25	*tet(M)*, *intTn*	tetracycline
		1	6	pS86	–	–
		2	6, 9	pCF10, pS86	–	–
		2	8, 9	pCF10, pAM373	*tet(M)*, *intTn*	tetracycline
		1	2, 6, 9	pCF10, pS86, pRE25,	*tet*(M), *erm*(B), *intTn*	tetracycline, erythromycin
		1	2, 7	pRE25, pUSA02	*tet*(M), *erm*(B), *intTn*	tetracycline, erythromycin
44	5	2	9	pCF10	–	–
		2	9	pCF10	*tet*(M), *intTn*	tetracycline
		1	6, 9	pCF10, pS86	*tet*(M)	tetracycline
55	3	1	2	pRE25,	*tet*(M), *intTn*	tetracycline
		1	2	pRE25,	*tet*(M), *erm*(B), *intTn*	tetracycline, erythromycin
		1	2, 8	pRE25, pAM373	*tet*(M), *intTn*	tetracycline
56	5	4	9	pCF10	–	tetracycline
		1	6, 9	pCF10, pS86	–	tetracycline
59	1	1	9	pCF10	–	–
62	1		–	–	–	–
63	1		9	pCF10	*tet*(M), *intTn*	tetracycline
64	2	1	9	pCF10	*tet*(M), *intTn*	tetracycline
		1	9	pCF10	*tet*(M), *erm*(B), *intTn*	tetracycline, erythromycin, trimethoprim
72	5	1	6	pS86	–	–
		1	9	pCF10	–	–
		3	6, 9	pCF10, pS86	–	–
79	1		6	pS86	–	–
81	3	1	–	–	–	–
		1	9	pCF10	–	–
		1	6, 9	pCF10, pS86	–	tetracycline
91	1		9	pCF10	–	–
97	2	1	–	–	–	–
		1	9	pCF10	–	–
105	1		2, 7	pRE25, pUSA02	*erm*(B)	erythromycin
162	4		9	pCF10	–	–
170	1		9	pCF10	*tet*(M), *intTn*	tetracycline
205	1		–	–	–	–
206	2	1	9	pCF10	–	–
		1	6	pS86	*tet*(M), *intTn*	tetracycline
209	4	1	–	–	–	–
		2	9	pCF10	–	–
		1	6, 9	pCF10, pS86	–	–
220	1		9	pCF10	–	–
226	1		–	–	–	–
236	2		9	pCF10	–	–
237	1		9	pCF10	–	–
238	1		6, 9	pCF10, pS86	–	–
239	1		6, 9	pCF10, pS86	–	–
240	1		9	pCF10	–	–
241	2	1	–	–	–	–
		1	9	pCF10	–	tetracycline
242	1		9	pCF10	–	–
243	1		9	pCF10	–	–
244	1		2, 8	pRE25, pAM373	*tet*(M)	tetracycline
245	1		9	pCF10	–	–
246	1		9	pCF10	*tet*(M), *erm*(B), *intTn*	tetracycline, erythromycin, trimethoprim
247	1		9	pCF10	–	–

* Sequence type and resistance obtained from our previous study [6].

**Figure 1:**
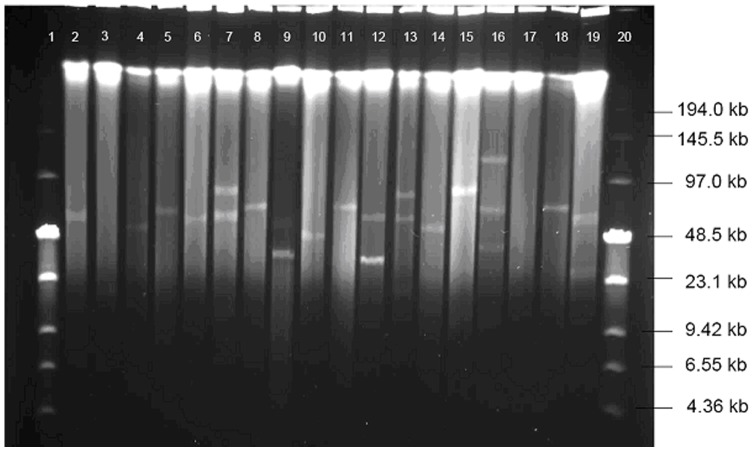
S1-PFGE of plasmid content. Lane1 and 20: Low Range PFG Marker, lane2–17: sample strains 9–24, lane 18: *E. faecalis* OG1X harbouring pCF10 (67.7 kb), lane 19: *E. faecalis* DS16 harbouring pAD1 (58 kb) and pAD2 (25 kb).

PFGE of S1-nuclease digested total enterococcal DNA allows detection and size estimation of plasmids as they appear as linearized bands of different sizes in a faint genomic background [23]. A total of 145 plasmid bands were identified in 95 (89.6%) *E. faecalis* strains by S1-PFGE (an example is shown in Figure 1). Among the plasmid positive strains, 59 carried one plasmid, 27 carried two plasmids, five carried three, three carried four, and one strain carried five plasmids. Plasmid sizes varied between 5–150 kbp.

Table 2 reveals comparative data obtained from plasmid *rep*-typing and S1-PFGE. Plasmids were detected in 85 strains by both S1-PFGE and PCR *rep*-typing, in ten strains only by S1-PFGE, and in eight strains by PCR alone. Statistical analysis revealed a significant correlation between the number of PCR-positive replicon and the plasmid number identified by S1-PFGE (*p* = 0.005). Table 3 shows the sequence identity of replicon PCR products by comparison with the respective *rep*-family control sequences in each *rep*-family.

**Table 2:** Plasmid identification by S1-PFGE and replicon typing.

Typing method	Strain number (N = 106)	Plasmid			
		Total number	Number per strain		Size range*(kb)
			Range	Mean±SD	
S1-PFGE	95 (89.6%)	145	0–5	1.4±0.90	5–150
*Rep-*typing	93 (87.7%)	120	0–3	1.1±0.63	4.4–67.7

* S1-PFGE plasmid size range based on our experimental data. *Rep*-typing size range based on published sequences.

**Table 3:** Sequence identity of replicons detected by PCR *rep*-typing in oral *E. faecalis* by comparison with the respective *rep*-family control sequence.

*rep*-family	Total number of replicon detected	Number of replicon sequenced	Sequence identity (%)
*rep*1	1	1	97.4%
*rep*2	8	8	100
*rep*6	22	11	100
*rep*7	2	2	93.7%
*rep*8	4	4	95.9–98.7
*rep*9	81	40	84.5–97.9%
*rep*17	1	1	100

### Detection of genes usually present on Tn*916*-like elements

The detection of genes usually present on Tn*916-like* elements (often found on pCF10-like plasmids) among *E. faecalis* is summarized in Table 1. The two genes (*tet*(M) and *intTn)* often present on Tn*916*-like transposon were detected in 33 *E. faecalis* strains and three genes (*tet*(M), *erm*(B) and *intTn*) often present on Tn*1545*-like transposon were detected in eight strains. Most of these strains were positive for *rep*9 plasmids by PCR. All *rep*2-positive strains were resistant to tetracycline and/or erythromycin, and six of them contained Tn*916*/Tn*1545* genes.

## Discussion

The present study investigated the occurrence, classification and characterization of plasmids in a national collection of *E. faecalis* isolated from subgingival dental plaque of patients with marginal periodontitis by two plasmid typing methods, PCR based *rep*-typing and S1-PFGE.

Recently a classification system has been proposed for identifying plasmids from Gram-positive bacteria by PCR-based *rep*-typing method [16]. The system is set up on PCR amplification of conserved regions of the replication initiation genes of plasmids. S1- PFGE is a classical method where plasmid DNA is linearized by S1 restriction enzyme, and separated on a gel to estimate their sizes [20], [23]. Based on the assumption of each plasmid replicon representing one plasmid by PCR *rep*-typing and each plasmid band representing one plasmid by S1- PFGE, 93 (87.7%) plasmid-positive strains were identified by PCR *rep*-typing and 95 (89.6%) plasmid-positive strains identified by S1-PFGE. Rosvoll et al. detected plasmid replicons in 83% of clinical *E. faecium* strains [20] and Jensen et al. reported a prevalence of 68% *E. faecalis* strains from both non-human and human origin carrying replicons [16]. These plasmid prevalence rates indicate that most *E. feacalis* and *E. faecium* strains contain plasmids with an amplifiable *rep* gene by PCR. The present study identified a total of seven *rep*-families in oral *E. faecalis* isolates, while Jensen and his co-workers detected ten *rep*-families from 28 *E. faecalis* and 51 *E. faecium* strains with the same method [16]. This finding agrees with the fact that numerous types of plasmids are often present in clinical enterococci [24], [25], [26]. It has been suggested that antimicrobial resistant flora may lead to recurrence and progression of periodontital diseases [27], [28]. Different plasmids being identified in high frequency and large numbers from the subgingival *E. faecalis* implicates oral enterococci might be a potential source of transferable antimicrobial resistance, as well as play a role in recurrent marginal periodontitis. In S1-PFGE experiments we detected plasmids ranging from 5–150 kbp in size and as many as five distinct plasmids in one strain. Accordingly other studies have shown that single isolates of *E. faecalis* may harbour multiple plasmids with different sizes and copy numbers [25], [29].

*Rep*9 (pCF10) was the most predominant *rep-*family among the oral *E. faecalis* with half of the strains in this group being resistant to tetracycline and three were resistant to erythromycin. *Rep*9 consists of five sex-pheromone responsive plasmids that are often found in *E. faecalis*. Sequence identity among this family was 81.8% [16] which corresponds to our finding of 84.5%–97.9% when compared to *rep*9 control sequence. This big sequence divergence most likely reflects the specificity of plasmids respond to different sex pheromones [16]. Comparing *rep*9 and MLST sequence types, a significant correlation is found between *rep*9 plasmids and ST21. For *E. faecalis* ST21 strains carrying *rep*9 plasmids, 17/19 of the strains were resistant to tetracycline. Plasmid pCF10 is a major member of *rep*9 that contains *tet*(M) on Tn*916-*like elements. It is likely that the majority of the tetracycline resistance is due to Tet(M) encoded by plasmid located *tet*(M) although this requires experimental verification. It is worth noting however that some isolates carrying *rep*9 plasmids do not exhibit tetracycline or erythromycin resistance, which could be indicative of variability of the plasmids in these strains in terms of association with Tn*916*-like conjugative transposons. Likewise a few resistant isolates had no detectable plasmids indicative of chromosomally located resistance genes [30], [31]. *Rep*2 (pRE25) was detected in eight strains. Interestingly all *rep*2-positive strains were resistant to tetracycline and/or erythromycin, and six of them contained Tn*916*/Tn*1545* genes. *Rep*2 family consists of six members that are so far known to exist in *Enterococcus* genus. pRE25 is a major member of this family that was first obtained from *E. faecalis* RE25 [32]. Resent studies demonstrate that the *rep*2 plasmids from enterococci can confer multiple antibiotic resistance [16], [20], [33] as well as toxin*-*antitoxin plasmid stabilization mechanism [20], [32].

Comparing PCR-based *rep*-typing and the S1-PFGE assay, we could see a statistically significant correlation between plasmid numbers detected by the two methods. This correlation indicates the two methods have similar discriminatory power for plasmid identification in *E. faecalis*. However, a higher number of plasmids and more plasmid-positive strains were screened by S1-PFGE. A possible explanation for this discrepancy could be that *rep*-typing will miss novel plasmid replicons, or those replicons which have diverged enough from the primer consensus sequence, and those plasmids which are not included in the PCR *rep*-typing system. Therefore the *rep*-typing should be extended continuously to cover other known and new replicon types. A potential source of new plasmids is our isolates where plasmids are detected by S1-nuclease PFGE alone; however this requires further investigation in order to determine the exact nature of these plasmids and is beyond the scope of this study. Due to the ability of plasmids to acquire a large repertoire of inserted elements, such as insertion sequences, transposons, integrons, and gene cassettes, it still remains a challenge for establishing a good system for plasmid identification and classification.

In summary, diverse plasmids have been identified in high frequency and in large numbers from the clinical strains of *E. faecalis* from patients with periodontitis by PCR *rep*-typing and S1-PFGE. An association was observed between *rep*9 plasmids and MLST ST21 strains, as well as between *rep*2 plasmids and Tn*916*-like elements and tetracycline and/or erythromycin resistance. The results of this dual analysis suggest that *E. faecalis* strains of periodontal infections carry multiple plasmids within different *rep*-families and they could be a potential source of transferable antimicrobial resistance. It also demonstrates that either S1-PFGE or plasmid *rep*-typing is currently not sufficient on its own to detect all types of plasmids in a group of isolates.

## References

[1] Ready D, Pratten J, Roberts AP, Bedi R, Mullany P, et al (2006) Potential role of *Veillonella spp*. as a reservoir of transferable tetracycline resistance in the oral cavity. Antimicrob. Agents Chemother 50: 2866–2868. 10.1128/AAC.00217-06 PMC1538667 16870789

[2] Roberts AP, Mullany P (2010) Oral biofilms: a reservoir of transferable, bacterial, antimicrobial resistance. Expert Rev Anti Infect Ther 8: 1441–1450. 21133668 10.1586/eri.10.106

[3] Villedieu A, Diaz-Torres ML, Hunt N, McNab R, Spratt DA, et al (2003) Prevalence of tetracycline resistance genes in oral bacteria. Antimicrob. Agents Chemother 47: 878–882. 10.1128/AAC.47.3.878-882.2003 PMC149302 12604515

[4] Villedieu A, Diaz-Torres ML, Roberts AP, Hunt N, McNab R, et al (2004) Genetic basis of erythromycin resistance in oral bacteria. Antimicrob. Agents Chemother 48: 2298–2301. 10.1128/AAC.48.6.2298-2301.2004 PMC415603 15155239

[5] Sun J, Song X (2011) Assessment of antimicrobial susceptibility of *Enterococcus faecalis* isolated from chronic periodontitis in biofilm versus planktonic phase. J Periodontol 82: 626–631. 21054225 10.1902/jop.2010.100378

[6] Sun J, Song X, Kristiansen BE, Kjaereng A, Willems RJ, et al (2009) Occurrence, population structure, and antimicrobial resistance of enterococci in marginal and apical periodontitis. J Clin Microbiol 47: 2218–2225. 19420168 10.1128/JCM.00388-09 PMC2708501

[7] Dahlen G, Samuelsson W, Molander A, Reit C (2000) Identification and antimicrobial susceptibility of enterococci isolated from the root canal. Oral Microbiol Immunol 15: 309–312. 11154422 10.1034/j.1399-302x.2000.150507.x

[8] Pinheiro ET, Gomes BP, Drucker DB, Zaia AA, Ferraz CC, et al (2004) Antimicrobial susceptibility of *Enterococcus faecalis* isolated from canals of root filled teeth with periapical lesions. Int Endod J 37: 756–763. 15479258 10.1111/j.1365-2591.2004.00865.x

[9] Rams TE, Feik D, Young V, Hammond BF, Slots J (1992) Enterococci in human periodontitis. Oral Microbiol Immunol 7: 249–252. 1408361 10.1111/j.1399-302x.1992.tb00034.x

[10] Hegstad K, Mikalsen T, Coque TM, Werner G, Sundsfjord A (2010) Mobile genetic elements and their contribution to the emergence of antimicrobial resistant *Enterococcus faecalis* and *Enterococus faecium* . Clin Microbiol Infect 16: 541–554. 20569265 10.1111/j.1469-0691.2010.03226.x

[11] Hollenbeck BL, Rice LB (2012) Intrinsic and acquired resistance mechanisms in *enterococcus* . Virulence 3: 421–433. 23076243 10.4161/viru.21282 PMC3485979

[12] Roberts AP, Mullany P (2011) Tn*916*-like genetic elements: a diverse group of modular mobile elements conferring antibiotic resistance. FEMS Microbiol Rev 35: 856–871. 21658082 10.1111/j.1574-6976.2011.00283.x

[13] Clewell DB, Francia MV, Flannagan SE, An FY (2002) Enterococcal plasmid transfer: sex pheromones, transfer origins, relaxases, and the *Staphylococcus aureus* issue. Plasmid 48: 193–201. 12460535 10.1016/s0147-619x(02)00113-0

[14] Gazzola S, Fontana C, Bassi D, Cocconcelli PS (2012) Assessment of tetracycline and erythromycin resistance transfer during sausage fermentation by culture-dependent and -independent methods. Food Microbiol 30: 348–354. 22365347 10.1016/j.fm.2011.12.005

[15] Smalla K, Osborn M, Wellington EMH (2000) Isolation and characterisation of plasmid from bacteria. In: Thomas CM editor. The horizontal gene pool: Bacterial plasmids and gene spread. harwood Academic Publishers, Amsterdam. pp. 207–248

[16] Jensen LB, Garcia-Migura L, Valenzuela AJ, Lohr M, Hasman H, et al (2010) A classification system for plasmids from enterococci and other Gram-positive bacteria. J Microbiol Methods 80: 25–43. 19879906 10.1016/j.mimet.2009.10.012

[17] Creti R, Imperi M, Bertuccini L, Fabretti F, Orefici G, et al (2004) Survey for virulence determinants among *Enterococcus faecalis* isolated from different sources. J Med Microbiol 53: 13–20. 14663100 10.1099/jmm.0.05353-0

[18] Sedgley CM, Molander A, Flannagan SE, Nagel AC, Appelbe OK, et al (2005) Virulence, phenotype and genotype characteristics of endodontic *Enterococcus* spp. Oral Microbiol Immunol 20: 10–19. 15612939 10.1111/j.1399-302X.2004.00180.x

[19] Carvalho MdGS, Steigerwalt AG, Morey RE, Shewmaker PL, Teixeira LM, et al (2004) Characterization of Three New Enterococcal Species, *Enterococcus* sp. nov. CDC PNS-E1, *Enterococcus* sp. nov. CDC PNS-E2, and *Enterococcus* sp. nov. CDC PNS-E3, Isolated from Human Clinical Specimens. J Clin Microbiol 42: 1192–1198. 15004074 10.1128/JCM.42.3.1192-1198.2004 PMC356851

[20] Rosvoll TC, Pedersen T, Sletvold H, Johnsen PJ, Sollid JE, et al (2010) PCR-based plasmid typing in *Enterococcus faecium* strains reveals widely distributed pRE25-, pRUM-, pIP501- and pHTbeta-related replicons associated with glycopeptide resistance and stabilizing toxin-antitoxin systems. FEMS Immunol Med Microbiol 58: 254–268. 20015231 10.1111/j.1574-695X.2009.00633.x

[21] Gevers D, Masco L, Baert L, Huys G, Debevere J, et al (2003) Prevalence and diversity of tetracycline resistant lactic acid bacteria and their tet genes along the process line of fermented dry sausages. Syst Appl Microbiol 26: 277–283. 12866855 10.1078/072320203322346137

[22] Ng LK, Martin I, Alfa M, Mulvey M (2001) Multiplex PCR for the detection of tetracycline resistant genes. Mol Cell Probes 15: 209–215. 11513555 10.1006/mcpr.2001.0363

[23] Tenover FC, Arbeit RD, Goering RV, Mickelsen PA, Murray BE, et al (1995) Interpreting chromosomal DNA restriction patterns produced by pulsed-field gel electrophoresis: criteria for bacterial strain typing. J Clin Microbiol 33: 2233–2239. 7494007 10.1128/jcm.33.9.2233-2239.1995 PMC228385

[24] Zhu W, Murray PR, Huskins WC, Jernigan JA, McDonald LC, et al (2010) Dissemination of an *Enterococcus* Inc18-Like vanA Plasmid Associated with Vancomycin-Resistant *Staphylococcus aureus* . Antimicrob Agents Chemother 54: 4314–4320. 20660665 10.1128/AAC.00185-10 PMC2944587

[25] Garcia-Migura L, Sanchez-Valenzuela AJ, Jensen LB (2011) Presence of glycopeptide-encoding plasmids in enterococcal isolates from food and humans in Denmark. Foodborne Pathog Dis 8: 1191–1197. 21793656 10.1089/fpd.2011.0897

[26] Rosvoll TCS, Lindstad BL, Lunde TM, Hegstad K, Aasnæs B, et al (2012) Increased high-level gentamicin resistance in invasive *Enterococcus faecium* is associated with aac(6′)Ie-aph(2″)Ia-encoding transferable megaplasmids hosted by major hospital-adapted lineages. FEMS Immunol Med Microbiol 66: 166–176. 22672387 10.1111/j.1574-695X.2012.00997.x

[27] Handal T, Caugant DA, Olsen I (2003) Antibiotic resistance in bacteria isolated from subgingival plaque in a norwegian population with refractory marginal periodontitis. Antimicrob Agents Chemother 47: 1443–1446. 12654689 10.1128/AAC.47.4.1443-1446.2003 PMC152519

[28] Soares GM, Figueiredo LC, Faveri M, Cortelli SC, Duarte PM, et al (2012) Mechanisms of action of systemic antibiotics used in periodontal treatment and mechanisms of bacterial resistance to these drugs. J Appl Oral Sci 20: 295–309. 22858695 10.1590/S1678-77572012000300002 PMC3881775

[29] Sedgley C, Clewell DB (2004) Bacterial plasmids in the oral and endodontic microflora. Endodontic Topics 9: 37–51.

[30] Murray BE (1998) Diversity among multidrug-resistant enterococci. Emerg Infect Dis 4: 37–47. 9452397 10.3201/eid0401.980106 PMC2627656

[31] Manson JM, Hancock LE, Gilmore MS (2010) Mechanism of chromosomal transfer of *Enterococcus faecalis* pathogenicity island, capsule, antimicrobial resistance, and other traits. Proc Natl Acad Sci 107: 12269–12274. 20566881 10.1073/pnas.1000139107 PMC2901427

[32] Schwarz FV, Perreten V, Teuber M (2001) Sequence of the 50-kb conjugative multiresistance plasmid pRE25 from *Enterococcus faecalis* RE25. Plasmid 46: 170–187. 11735367 10.1006/plas.2001.1544

[33] Palmer KL, Kos VN, Gilmore MS (2010) Horizontal gene transfer and the genomics of enterococcal antibiotic resistance. Curr Opin Microbiol 13: 632–639. 20837397 10.1016/j.mib.2010.08.004 PMC2955785

